# Science and Healthy Meals in the World: Nutritional Epigenomics and Nutrigenetics of the Mediterranean Diet

**DOI:** 10.3390/nu12061748

**Published:** 2020-06-11

**Authors:** Fabio Caradonna, Ornella Consiglio, Claudio Luparello, Carla Gentile

**Affiliations:** Department of Biological, Chemical and Pharmaceutical Sciences and Technologies, University of Palermo, Viale delle Scienze, edificio 16, 90128 Palermo, Italy; ornella.consiglio@libero.it (O.C.); claudio.luparello@unipa.it (C.L.); carla.gentile@unipa.it (C.G.)

**Keywords:** nutrigenetics, nutrigenomics, Mediterranean diet

## Abstract

The Mediterranean Diet (MD), UNESCO Intangible Cultural Heritage of Humanity, has become a scientific topic of high interest due to its health benefits. The aim of this review is to pick up selected studies that report nutrigenomic or nutrigenetic data and recapitulate some of the biochemical/genomic/genetic aspects involved in the positive health effects of the MD. These include (i) the antioxidative potential of its constituents with protective effects against several diseases; (ii) the epigenetic and epigenomic effects exerted by food components, such as Indacaxanthin, Sulforaphane, and 3-Hydroxytyrosol among others, and their involvement in the modulation of miRNA expression; (iii) the existence of predisposing or protective human genotypes due to allelic diversities and the impact of the MD on disease risk. A part of the review is dedicated to the nutrigenomic effects of the main cooking methods used in the MD and also to a comparative analysis of the nutrigenomic properties of the MD and other diet regimens and non-MD-related aliments. Taking all the data into account, the traditional MD emerges as a diet with a high antioxidant and nutrigenomic modulation power, which is an example of the “Environment-Livings-Environment” relationship and an excellent patchwork of interconnected biological actions working toward human health.

## 1. Introduction

All over the world, the Mediterranean basin is famous for its historical architectural beauties and its agro-alimentary products. Within the international debate on a shift towards more sustainable food systems and diets, interest in the diet typical of the olive-growing areas of the Mediterranean basin (mainly Greece, Spain, Italy, and France) as a model of a sustainable dietary pattern has increased [1]. Sustainable diets are protective and respectful of biodiversity and ecosystems, culturally acceptable, accessible, economically fair and affordable, nutritionally adequate, safe, and healthy [2]. The Mediterranean diet (MD) represents the legacy of the link between man and the Mediterranean environment that has existed since the time of the last Ice Age [3]. In the Mediterranean basin, it is frequent that the distance between the source of food and the consumer is short, and thus today in these countries it is possible to distinguish the local food from global food. Local food is usually far fresher and without or with few preservatives or pesticide residues due to a non-chemical soil fertility and a nutrient-respectful cooking manner for the preservation of the phytochemicals [4]. In particular, the practice of using fresh seasonal ingredients is typical in Mediterranean cuisine. On the other hand, simply eating vegetables with extra virgin olive oil, for example, is the quintessential Mediterranean way of obtaining the benefits of both taste and health. Following this Mediterranean style, the food has a personal symbolic value (of pleasure), so “eating does not mean just feeding!”. In November 2010, the MD was inscribed in the UNESCO Representative List of the Intangible Cultural Heritage of Humanity.

Nutritional research on the health value of the MD has been certainly encouraged by a number of scientific results collected over the last fifty years that documented the decisive role that nutrition plays on human health. Based on the most recent studies in the nutritional field, despite the numerous dietary models continuously proposed, the MD—characterized by a high consumption of fruit, vegetables, and extra virgin olive oil and a moderate wine consumption—remains the qualitatively superior food style [5]. Numerous epidemiological studies have shown that populations following the MD have a higher life expectancy and a reduced incidence of various chronic diseases, such as cardiovascular and neurodegenerative diseases, with respect to Northern Europe and the USA [6,7]. It also appears that this food style is able to reduce the risk of developing various types of cancer [8] and is beneficial in the course of autoimmune diseases [9]. In addition, a more recent paper reports some beer-contained bioactive compounds (e.g., Xanthohumol, Silicon) as potential instruments for protecting against neurodegenerative diseases [10]. In fact, a moderate consumption of beer during main meals must also be recorded as a recent additional component of the MD [11].

Referring to epigenetic influences, the hypothesis that the MD could act as an environmental protection for the consumer just as pregnancy does for the fetus [12] is stimulating and likely.

## 2. Finality, Criteria, Scheme, and Drafting Method of This Review

This review describes the biochemical/genomic/genetic aspects involved in the positive health effects related to the MD with the aim to give a speculative/scientific explanation of its perceived beneficial properties under these specific aspects. For this goal, the MD is intended as that diet regimen in use in the Mediterranean basin, which is fundamentally made up of local fresh seasonal non-industrial ingredients with a nutrient-respectful cooking manner and composed of three meals a day with a very light breakfast, a lunch, and a dinner—the latter both articulated into three courses. For a greater punctual precision, we want to mainly refer to the MD components described by the 14 items included in the MEDAS PREDIMED nutritional intervention carried out in Spain from 2003 to 2011 [13], which was recently validated [14] and is currently used in nutritional studies with MD as a topic [15].

A precise bibliographic choice has been made as functional for the purpose of the review. Only articles that report nutrigenomic or nutrigenetic data were taken into consideration, avoiding those dealing with a general health effect without the involvement of the genome or its expression. In particular, as regards the nutrigenetic section, only those articles that help to put in raw (i) polymorphisms, (ii) the adoption of the Mediterranean diet, and (iii) the main related diseases were held in consideration.

For these purposes, only the Scopus and PubMed public repositories were used, choosing 2010 as the year limit; occasional previously published articles have rarely become necessary as a historical basis for a better understanding of recent findings. The search terms “nutrigenomic, nutrigenetic” and alternatively “epigenetic diet, polymorphism diet” were always used.

With these premises, we therefore attempt to describe the nutrigenomic and nutrigenetic value of the MD by pointing out that most of these nutrients and their specific biological effects are known only in specific bio-systems under specific conditions and are valid for the determined genetic profile, ethnicity, age, and gender.

## 3. Healthy Properties of Mediterranean Diet

The World Health Organization (WHO) estimates that about 30% of cancer deaths are attributed to modifiable risk factors such as eating habits, lifestyle, physical inactivity, smoking, and alcohol consumption [16]. On the other hand, there is epidemiological, clinical, and experimental evidence suggesting that the diet is one of the most important factors influencing human health. Indeed, an appropriate diet is not only important to avoid the onset of diseases due to nutrient excesses or deficiencies, preserving our health status, but it can actively contribute to our well-being. On the other hand, as established by the WHO in 1948, health is “A state of complete physical, mental and social well-being and not merely the absence of disease or infirmity”.

The evident link between diet and health has directed the interest of scientific research towards the study of the components responsible for the condition of well-being [17,18]. Apart from the nutritional significance of macronutrients in compensating for energy and material losses, the functional; protective; and, ultimately, “therapeutic” properties of certain foods or, more generally, of specific dietary patterns, depend on specific small food molecules with biological activity [19,20]. Sterols, bioactive peptides, polyunsaturated fat acids, vitamins, alkaloids, and polyphenols are included in this category. In particular, plants are the most important source of natural bioactive substances and still represent the main resource in the quest for new drugs. 

The MD is characterized by a favorable lipid profile for cardiovascular function, with a high percentage of unsaturated fatty acids from olive oil and nuts [21]. On the other hand, the MD, due to its high content in plant-derived foods, is an extraordinary source of phytochemicals and also ensures a high intake of fibers [22,23]. Key foods of the MD, such as olive oil, oily fish, nuts, yoghurt, several fruits, spices, and herbs, are considered functional foods due to their nutraceutical content [24]. Consequently, several MD foods for their high content of active constituents might be exploited in the formulation of nutraceuticals or for the functionalization of other foods [25].

Nuts are rich in unsaturated fatty acids, plant proteins, fiber, antioxidant vitamins, phenolic compounds, and salutary minerals [26,27]. It is currently acknowledged that nut consumption, by lowering serum concentrations of total and low-density lipoprotein cholesterol, reduces the risk of cardiovascular diseases (CVDs) [6]. Additionally, other studies suggest that some positive effects of the consumption of nuts can be correlated with their content of phytochemicals with antioxidant and antinflammatory properties [28,29].

Accumulating evidence suggests that extra virgin olive oil (EVOO), due its high content in unsaturated fatty acids and phytochemicals, may have several health benefits, including cardiovascular protection, cancer prevention, and antinflammatory properties [30].

The characteristic dairy products of the Mediterranean diet (MD), such as cheese and yoghurt, are better tolerated by lactose-intolerant individuals. In particular, yoghurt—the world’s most commonly consumed fermented milk—due to its live bacteria content can produce significant health benefits, including improvements in gastrointestinal health and immune response [31]. Moreover, due to the high consumption rate of yoghurt, its fortification could add an important nutritional value [32], and with this purpose the addition of different bioactive ingredients from plant, fungi, and algae extracts has been evaluated [33,34].

Herbs and spices used as condiments in the MD, due their high content of antioxidant phytochemicals, can greatly increase the nutraceutical value of a typical meal. The caper, Capparis spinosa L., is one of the most common aromatic plants spontaneously growing in the Mediterranean basin. This floral bud contains high levels of alkaloids, glucosinolates, and polyphenols, and is frequently consumed as an appetizer or as a complement of other foods, such as salads, pasta, meat, etc. [35]. Although the caper cannot be considered as a food with a very high antioxidant potential, experimental data show that caper extracts in very small amounts, consistent with the typical use of the caper buds as a cooking flavor, are effective in preventing lipid peroxidation in red meat during digestion [36].

### Antioxidative Potential of the Mediterranean Diet

Although the protective effect associated with the consumption of dietary phytochemicals is due to a wide diversity of mechanisms, it has been frequently linked to their ability to function as antioxidant molecules influencing the cellular redox balance and then contributing to the prevention of oxidative stress phenomena [37]. 

Many studies suggest that the MD is able to protect against oxidative stress [38]; in particular, it was demonstrated that a high adherence to the MD is positively correlated with the total antioxidant capacity of healthy subjects [39] and obese patients [40].

Although a large emphasis has been given to the low production of cooking-related oxidants due to a high consumption of raw foods typical of the MD [38,39], the positive effects of this dietary pattern on redox homeostasis are mainly related to the high content of antioxidant molecules. 

The cellular redox balance is the delicate equilibrium that is established between the production of oxidizing species and antioxidant defenses. The physiological cellular metabolism involves oxidative processes; it has been shown that, during ATP production, at least 1% of oxygen is not completely reduced, resulting in ROS production. On the other hand, ROS production can be induced by exposure to several chemical and physical agents. The production of oxidizing species is controlled under physiological conditions by endogenous antioxidant defense mechanisms, which include soluble components, such as glutathione and melatonin, and insoluble antioxidant defenses referable to antioxidant enzymes.

The deregulation of the subtle balance between oxidizing species and antioxidant defenses is the cause of cellular oxidative stress. Epidemiological data show that cellular oxidative damage is involved in the occurrence and progression of several chronic diseases, including cancer, inflammation, and neurodegenerative diseases. 

The intake of antioxidant molecules improves endogenous antioxidant defenses and contributes to preventing oxidative stress. Experimental data show that dietary antioxidants are not only able to neutralize reactive species through a direct scavenger action but also to modulate the expression and the activity of antioxidant enzymes [41].

It must also be emphasized that, due their ability to influence the cellular redox state, dietary antioxidants not only protect from oxidative stress phenomena but, through structural changes in redox-sensitive sites in the cell, they can also modulate the function of numerous biological targets at the key sites of signal transduction pathways [42] or DNA integrity [43]. 

**Figure 1:**
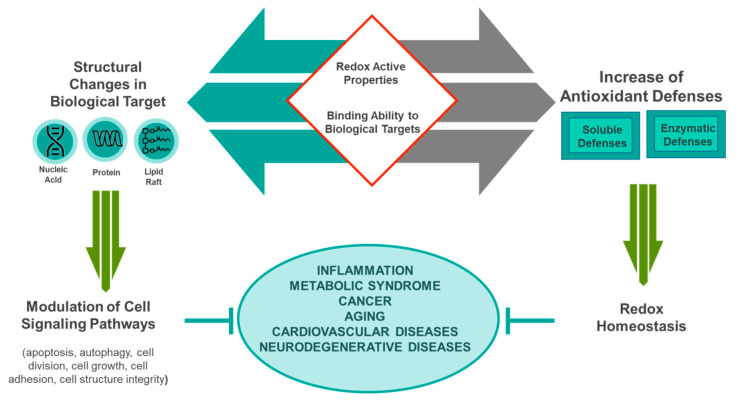
Biochemical mechanisms of phytochemical bioactivity.

On the other hand, in some cases, regardless of the active redox properties, a direct interaction of those small bioactive molecules with the target in the cell may be sufficient to influence the function of that target [44]. Among the large number of targets that a phytochemical can have in a cell, the interactions with DNA may be sufficient to influence its function with a lot of consequent actions and effects (Figure 1).

## 4. The Epigenomics of Mediterranean Nutrients

In a virtual “DNA-nutrient interactions” scheme, if we consider the nutrient as the protagonist and its relationship with DNA expression as the target, we are speaking about nutrigenomics, the modern -omic science that has had an explosion of interest in the last fifteen years.

Nutrigenomics describes, in particular for nutrients, the already-known epigenetic interactions between the environment and DNA. There are several actors in nutrient–DNA interactions: the knowledge of all their contributions is necessary to explain how food components, food, diet, and lifestyle can influence the trajectory toward the human health condition [45]. For instance, maternal nutrition during pregnancy represents a clear example of a critical environmental factor that is diet-inherent and conditioning the genome expression, and is finally a phenotype of the fetus which determines the risk of disease in adulthood [29].

Since it is widely known that all the -omic sciences are interdigitated in a unique “conversation”, the metabolomics, or better, the nutrimetabolomics, provides some biomarkers [45] which represent the best phenotype that nutrigenomics can study to understand the correspondent epigenetic modification. In this sense, the most recent term “foodomics” describes today the integrated face of nutrigenomics that makes use of the other -omic sciences to obtain a greater understanding of nutrient–DNA interaction phenomena.

Today, with the quantitative great number of specific literature, nutrigenomics can make a contributions to explain some cellular/molecular phenomena underlying human pathological conditions that can be adjuvated, prevented or better managed with the diet. In this sense, the MD is present in a big subset of this literature; it has been more precisely described in the last years and less precisely described in past ones, when a generic epigenetic hypothesis of the diet–pathology link was reported. For example, some years ago, high cortisol levels were found in the offspring of women with an alternate diet during their late pregnancy [46]. Subsequently, an unbalanced maternal diet in pregnancy was associated with offspring epigenetic changes in genes controlling the glucocorticoid action [47], and recently a correlation between the MD and cortisol levels at birth was reported [48], suggesting that a low MD adherence could exert epigenetic effects linked to modifications in fetal cortisol levels [29]. It is worth mentioning that a non-optimal hypothalamic axis activity, as measured by cortisol levels, has been identified as one potential risk factor for suicide or, more generally, mental diseases [49].

### 4.1. Mediterranean Foods/Drinks Contained Molecules Which Act as Epigenetic Modulators

Several bioactive molecules have since long been described as actors of pleiotropic actions in many diets, including the MD. At the “cellular” level, among the targets of the action by dietary bioactive molecules, DNA and chromatin assume the role of metabolic sensors [50]. In particular, recent data show that several dietary molecules can act as epigenetic modulators of (i) DNA methylation, (ii) histone acetylation/deacetylation, and (iii) small non-coding RNA action. Their ability to alter epigenetic patterns may depend on direct interaction with the enzymes responsible for adding or removing epigenetic marks or, indirectly, on the regulation of the expression of genes that encode proteins implicated in the epigenetic machinery [51]. In confirmation of this, the hypothesis can be reported that an altered mother nutrition and poor in dietary supply of methyl donors during pregnancy may result in permanent alterations in the DNA methylation patterns of newborns when the fetus epigenome is particularly responsive to environmental stimuli [52].

Fifteen years ago, some fruits and vitamin B12- and folate-containing vegetables gained the definition of antimutagens [53] and more recently acquired the functional description of methyl donors [54], being involved in the S-adenosine methionine (SAM) production/hydrolysis cycle. Coherently, a low adherence to the MD or a folate deficiency may cause LINE-1 hypomethylation in the blood leukocytes of healthy women and consequently genomic instability and cancer risk [55]. The MD, precisely, comprehends many folate-rich vegetables: asparagus, broccoli, artichokes, cauliflower, the famous little cherry tomatoes of Pachino (Ragusa, Sicily, Italy), and spinach. In particular, for the latter ones, it is important to report a study on an avian embryo model in which it was reported that supplementation with the folate-like methyl donor betaine naturally contained in spinach had even protected against the congenital defects induced by prenatal alcohol exposure, surely modulating epigenetic changes [56].

Moreover, the MD provides for the consumption of many typical fruits, all folate-rich: oranges, clementines, tangerines, strawberries, peaches, mangos, and red berries. In addition, other micronutrients, such as minerals important for the functionality of the main epigenetically acting enzymes (e.g., zinc, magnesium, and others), are also contained in MD foods like fruits, whole-meal cereals, nuts, and fish [57].

The fruit of the yellow cultivar Opuntia Ficus Indica, a typically two-seasonal MD fruit, deserves a separate discussion. It contains Indicaxanthin, a betalain that was described as a DNA methylation modulator at the genome and genic level. In Caco2 cells, in particular, this molecule is able to demethylate the genomic DNA and the promoter CpG islands of the P16^INK4A^ gene, with the consequent reactivation of the expression of this oncosuppressor gene [58]. It would be natural to correlate this specific and powerful effect of Indicaxanthin with the lower incidence of colon cancer attributed to MD consumers, but a specific correlation with in vivo studies is still lacking. Surely, this interesting molecule could be a good candidate as an anticancer epidrug, usable in combo-therapies.

Some dietary molecules positively influence the chromatin histone acetylation/deacetylation equilibrium. The molecule that gave coherent results, so much as to earn the definition an of anti-urologic tumor agent, is sulforaphane, a natural compound found in cruciferous vegetables from the Brassicaceae family such as broccoli, cauliflower, and cabbage, all copiously present in the MD. The molecular mechanism of action of this isothiocyanate is well explained by an epigenetic control of histone deacetylase (HDAC) activity. Animal daily sulforaphane consumption for three weeks suppressed the growth of human PC-3 prostate cancer cells by 40% in male nude mice and evoked a significant decrease in the HDAC activity in mononuclear blood cells, correlating well with tumor growth inhibition. In human subjects, a single dose of 68 g of broccoli sprouts significantly inhibited the HDAC activity in peripheral blood mononuclear cells 3 and 6 h following consumption [59]. Sulforaphane is today another interesting MD candidate for additive cancer treatment for its notable non-toxic anti-tumor effect showed in vitro and in vivo [60]. A recent idea that epigenetic alterations can lead to CVDs and that some dietary factors are able to counteract their actions has arisen, especially referring to the MD. Bioactive compounds such as resveratrol—present in grapes, nuts, berries, and red wine—may activate Sirtuins deacetylases, HDAC, or acetyltransferases. The MD with its foods and beverages can be a valid aid against CVDs, although the time in which these findings can become an easy dietary intervention for the prevention of or therapy for these diseases is still far off [61].

Many data suggest that bioactive food components and phytochemicals play a direct or indirect role in the modulation of miRNA expression [62,63,64], a new frontier in the understanding of tumorigenic mechanisms. In particular, in cancer it is possible to find some miRNAs that can act as onco-promoters and other ones that can act as onco-suppressors [65]. Our lab is generating some initial but interesting data in this field using one of the protagonists of the MD: extra virgin olive oil with its peculiar polyphenolic content, 3-Hydroxytyrosol (HT). We are aiming to highlight those miRNAs that are differently regulated in a small cohort of 50 patients with breast cancer after they have introduced into their diet two kind of EVOO, enriched, respectively, by 250 and 25 parts per million HT. By using RNA sequencing and the Open array with Taqman chemistry, we are analyzing the expression of 20 circulating miRNAs in comparison with these specific food interventions. As a preliminary result, we found that the increase in the expression of the onco-suppressor miR 15 and miR 16 in oncological patients seems to have a positive dose-dependent relation to the HT amount, suggesting that this diet regimen may have beneficial health effects in patients; similarly, the onco-promoters miR21 and miR1274a showed a rationally coherent variation, presenting lower expression levels in the patients who had taken HT-enriched oil (unpublished not-shown data). However, a more specific correlation between aging, age-related inflammation, circulating miRNAs, and diet is due soon [66] and needs further study.

Wine and cheese are typical foods in at least four countries of Mediterranean Europe (Greece, France, Spain, Italy). In the last 15 years, the “French paradox” phenomenon has summarized the observations of the low rates of CVDs and mortality in France despite the high saturated fat consumption of French people [67,68]. A non-negligible contribution to explaining this complex paradox could come from the nutrigenomic beneficial effects of red wine’s resveratrol; red wine very popular in France. While today there are controversial findings on resveratrol’s impact on human health [68], it was reported that this molecule may become an important anti-tumor agent against high-grade gliomas resistant to conventional therapies [69], probably also because of its putative property in regulating miR-21 expression [70]. Moreover, since recent studies have been mainly centered on the healthy effects of the dairy products largely consumed by the French population [71] but also by the Greek, Spanish, and Italian ones, we can hypothesize that this food could also have some beneficial nutrigenomic effects. The specific research on this topic using human sources and models is very poor. A recent study performed on dairy cattle described the effects of butyrate on ruminant epithelium transcriptome with the involvement of the cell cycle via histone modification and the consequent epigenetic regulation of gene expression [72]; this result encourages us to investigate in this direction for humans, too. Today, with the data in our possession, it is surely possible to say that the consumption of wine and cheese together, seen in nutrigenomic terms, could add precious information about the diet–DNA interaction of at least these two MD components.

A typical dish in Spain is known to be Paella, a combination of meat, fish, seafood, vegetables (peas, artichokes, carrots, beans), and spices. In fact, there is more than one type of Paella just containing only one type of meat (rabbit, pork), one type of fish, or only vegetables, but the most common is the one that contains a mix of all the aliments previously reported.

The meat, in particular chicken and pork, was reported to increase the metabolism of glutathione, the principal liver antioxidant defense system, following an extensive in vivo RNA seq study [73,74]. In absence of literature data about the specific nutrigenomic effects exerted by seafood, except for the known supply of Omega-3 polyunsaturated fatty acids (n-3 PUFA) [75], we can report that fish intake is capable of reducing the expression of genes responsible for cholesterol biostynthesis, producing a hypocholesterolemic effect in rats [73]. Moreover, the finding that concentrated pea saponins show a remarkable nutrigenomic effect on Atlantic salmon gut cells [76], in the absence of data on humans or on human cells, represents a promising research line which can be performed also in human models. Similar to these studies, in humans or human cells several genes could also be up or down-regulated by this pea component, and this influence could modulate inflammation, detoxification, DNA repair, and other systemic or cellular endpoints.

An extract of the edible part of fresh artichoke (*Cynara scolymus* L.) has been reported to cause an increased ROS production and cell death in several cancer cell lines and leukaemia cells, probably through a multiple epigenetic effect. In fact, it was demonstrated that this extract lowered the genome-wide DNA methylation and increased the level of lysine acetylation in most of the tested cell lines [77].

Paella is seasoned with spices, some of which are reported to have nutrigenomic effects, but not always in humans or human cells. Garlic and holy basil leaves enhance the immune response in birds, increasing the mRNA transcription for some Toll-like receptors [78]. Curcuma down-regulates the TNF, CXCL8, NFKB1, and PTGS2 genes, corroborating the anti-inflammatory action in dogs [79]. A polysaccharide from the peel of the Korean citrus hallabong enhances the NK cell function and attenuates the pro-inflammatory cytokine IL-12 and IFN-γ levels in a placebo-controlled study [80]. Moreover, a significant anticancer effect of clove buds in a mammary carcinoma model in vivo and in vitro has been documented as being capable of modulating histone modification [81] and DNA methylation [82]. Additionally, saffron may lead to the inhibition of cancer cell proliferation or/and the induction of apoptosis through various mechanisms, including the inhibition of DNA and RNA synthesis, interaction with cellular topoisomerase, suppression of telomerase activity, and targeting of microtubules [83].

Many fruits, with a few exceptions, are known to be “alkalinizers” and thus act against nephrolithiasis [84], the most common urinary disease with the formation of stones in the kidneys. A particular explanation is needed for dietary vinegar, due to its recently reported properly nutrigenomic effect [85]. Zhu and colleagues examined the in vitro and in vivo anti-nephrolithiasis effects of vinegar and acetate, suggesting that the latter enhanced the acetylation of H3 histone in renal tubular cells and promoted the expression of some microRNAs which reduce urinary calcium excretion. Thus, vinegar consumption is a promising strategy to prevent nephrolithiasis, thanks to its epigenetic effect.

Epigallocatechin has been widely described as an epigenetic modulator and is a component of some MD fruits, such as as cranberries and pomegranates [86,87]; it has been even proposed as an epigenetic adjuvant in Down Syndrome management to balance the abnormal DNA methlyation caused by the overdose of chromosome 21 genes [88]. Epigallocatechin is also able to regulate the expression of some important genes, among which are those encoding phase II antioxidant enzymes, DNA methyltransferases, and histone acetyl transferases: all these genes are capable of triggering/mediating numerous crucial epigenetic mechanisms in cells [89].

In Table 1, a synoptic list of some other typical MD foods (not described in the text) and their respective nutrigenomic effects is shown. Especially for the typical MD salad, since all the components are placed in a vertical order, it is possible to easily observe how large the summative nutrigenomic effect of an MD composite food can be.

### 4.2. Main MD Cooking Methods and Their Implications in Variations of Nutrigenomic Effects

It is quite understandable that the cooking method of a food has an important modulating role in the bioactivity of the phytochemicals contained and ultimately heavily influences their final effects on the organism, especially in nutrigenomic terms. For example, some methods add fats; solubilize, dilute, or disperse phytochemicals; or bring phytochemicals to high temperatures, making them unable to exert any epigenetic effects. Other methods are able to increase the concentrations of some biomolecules by depleting the food of aqueous content. Cooking, for humanity, has been and is a huge acquisition, if only for the sterilization and deactivation of some toxins contained in food, but it is necessary to profoundly analyze the various methods to understand, in the balance of risks/benefits, what is sacrificed and what is advantageous when we choose one cooking method over another. In confirmation, a very different profile of polyphenols was detected in food after gastrointestinal digestion in relation to how it was (un)cooked: raw, boiled, microwaved, grilled, fried in olive oil, fried in soybean oil [102].

With a similar reasoning, it is possible to associate a diet which traditionally uses some cooking methods more frequently than others with an appropriate healthy expectation and also in nutrigenomic terms. In the traditional MD, the main cooking methods are (i) boiling, (ii) frying, (iii) roasting, (iv) grilling, and (v) baking. With regards to nutrigenomic effects, validated data with identical analytical parameters before and after cooking, with a rigorous check of the relationship between the dried raw material and the dried prepared meal, are mainly present, nowadays, only for boiling and frying.

According to the traditional MD boiling method, a quantity of water is brought to the boil that is greater than the content of the vegetables. Once boiling is reached, the vegetables are added for a time that varies from a few minutes for leafy vegetables to about 20 min for legumes. The method of boiling the MD vegetables for 10 min was evaluated in terms of the conservation of phytochemicals. Boiling has been found to reduce bioactive compounds differently in relation to their chemical composition and plant source: phenolic compounds have been found to be more resistant to the boiling process, with the exception of vitamin C, while the content of antioxidants has been found to be decremented. Broccoli and lettuce were among the plants most affected by boiling, while cabbage and tronchuda cabbage maintained their biomolecule content [103]. In this regard, it could be interesting to report that a typical MD custom of obtaining a soup by boiling is to leave the legumes to hydrate overnight in a bowl full of water before cooking; in this way, the boiling times are shorter (about 10 min), and probably the phytochemicals and all the related nutrigenomic effects are more preserved.

Frying exclusively with EVOO, one of more appreciated and tasty MD cooking manners, is demonstrated to be a thermal treatment that contributes to release of nutrients from foods [104]. Moreover, when frying at a low temperature for a short time, only 9% of phenolic alcohols decreased [105]. Additionally, when frying with an EVOO obtained from olive fruits of minor ripeness, an increased stability of the oils was shown, probably due to a better fatty acid profile [106]. In fact, the Mediterranean tradition decrees that the olives collected are not quite ripe and that the frying with EVOO does not extend for more than 15–20 min at relatively low temperatures; therefore, most of the nutrigenomic powers associated with the polyphenolic components remains still active. Moreover, sofritos, a typical MD chopped mix of onions, carrots, and parsley fried in a pan with a low amount of EVOO for a few minutes, provides a substrate with additional bioactive compounds that can be added to soups, tomato sauce, and meat-enriched derivatives or used alone as a food garnish; in this way, in addition to a more pleasant taste, a summative quality and quantity of healthy compounds will be eaten, and there are several reasons to think that an extra nutrigenomic effect can be obtained. For instance, the positive effects of onion on the total lycopene bioaccessibility was hypothesized via its Z-isomerization during the heating of a tomato-based puree [107].

Lastly, it could be very interesting to look at the most-used cooking modes and evaluate their effect on the residual pesticide presence in cooked foods that could silence some nutrigenomic effects; unfortunately, these particular data are less present in the literature. Today, we are all at risk of having a chronic and daily low-dose exposure to these contaminants and, knowing that a particular cooking mode can increase or eliminate this damage, this could have a great public utility. In a retrospective view, it is also possible to highlight the main typical cooking mode of a popular diet regimen to assign an additional specific health score. Recently, an interesting study showed that chafing dish and soup methods, by dispersing vegetable content in the media, greatly lowered the pesticide residues, while to stir-frying method, conversely, increased them. Salad, in which no cooked vegetable is present, because of all the pesticide left preserved is a dish with a high risk [108]. Regarding the MD, it is worth remembering that frying with EVOO is a typical way of cooking and that the vegetables used for salad are often local, not coming from intensive farming, and are likely free of pesticides. However, it is also true that just as some cooking methods eliminate/concentrate pesticides, they could equally/differently do so with beneficial bioactive molecules. Although little general data about this topic are present in the literature [105], it will be therefore important to assess the possible selective capacity of some cooking methods and perform in vitro experiments with extracts from cooked foods to establish the residual nutrigenomic power of the molecules remaining bioactive after cooking.

## 5. Genetic Variants, the Mediterranean Diet, and Human Health

Similarly to the transition from pharmacology to pharmacogenetics, the genetics of food metabolism some years ago assumed the dignity of a specific science, nutrigenetics, also confirmed by the birth in 2005 of the International Society of nutrigenetics/nutrigenomics (https://www.nutritionandgenetics.org) and by the holding of the conference “2018 American College of Nutrition Meeting” [109]. For both sciences, the value of genetic variability, which is not at all negligible today, has been finally recognized; for too long, this fundamental concept was not considered even in scientific environments much more inclined to rely on the average individual, who does not exist, than on the genetic diversity expressed in the single person with his single genome. 

It is important to underline that in nutrigenetics it is more difficult than in conventional genetics to find a direct correspondence between the genotype and phenotype. In fact, taking, for example, “food wellness” as a specific phenotype, which is in itself a vague but useful phenotype, hundreds of genes and genotypes can contribute to its manifestation. Furthermore, if we also take into account the phenomenon of penetrance, in population studies, we risk being undetermined with each data report. The nutrigenetic studies accepted by the scientific community and present in the official literature are often based on assumptions that in most cases are true; for example, the contribution of a single gene (or single pathway) product(s) in a specific metabolism, revealed by a specific metabolic parameter and a range of penetrance that make evenly evident the manifestation object of the research.

Nutrigenetics has interconnections with at least three scientific disciplinary sectors: genetics, biochemistry, and physiology. Today, the power of massive sequencing techniques gives us the ability to describe specific allele contributions in the Mediterranean basin in relation to autochthonous people’s wellness.

### 5.1. Predisposing/Protective Genotypes, Main Human Diseases and the Mediterrranean Diet

In general, for the definition of a precision nutrition [110] it would be desirable that every individual were genotyped for all the related genes in order to establish an integrated individual gene profile useful to advise, by apposite software, the right diet in relation to his/her age and particular physiological conditions, such as sports stress, pregnancy, or others. In particular, one wonders if the MD may be able to lower, over time, the consequences of some known risky genotypes.

Obesity is a worldwide social-economic problem, and the MD is a potential collection of several food lifestyles against it. For this purpose, particularly interesting is the comparative analysis of three polymorphisms in the FTO (rs1558902 and rs9939609) and TCF7L2 (rs7903146) genes and their respective alleles, A and T [111]. Extrapolating these results, we could hypothesize that individuals carrying the FTO AA genotype, if they live in the Mediterranean area and adopt the MD, may show better results since the carriers of the FTO A allele are more prone to weight loss through diet or lifestyle interventions [112]. Another study [113] seems to confirm this hypothesis. In fact, individuals with the so-called “minor” FTO alleles (rs9939973, rs8050136, rs1781749, and rs3751812), different from those described by Nasreddine [111], had a lower risk of obesity when adopting the MD as a food lifestyle. Moreover, the FTO polymorphism (rs9939609) is also studied in an interesting research addressing the very complex relation between (i) the maternal genotype, (ii) mother diet during pregnancy, and (iii) newborn genotype for the onset of insulin resistance and CVDs. This study hypothesized that a high MD adherence during pregnancy was able to counterbalance the potential negative effects on glucose homeostasis in neonates independently from both the mothers’ and their own genotypes [48]. This suggests that adherence to a healthy diet, such as MD, can minimize the negative effects of a risky genotype although in a relatively short but important time such as pregnancy.

There is a great debate around the beneficial effects of the MD against coronary diseases, probably due to the great heterogeneity of the term “beneficial effects”. Even from the large and extensive comparative study in topic, it can be only concluded that there is still some uncertainty regarding the effects of a Mediterranean-style diet on the clinical endpoints of these disorders [114]. Despite these uncertainties, an interesting gene–diet interaction regarding the APOE gene and coronary diseases is described in long-term MD consumption. In particular, T-allele carrier MD consumers showed a more significant decrease in postprandial triglycerides and large triacylglycerol-rich lipoproteins in comparison with CC subjects; these results offer more precise dietary advice for these patients [115] and add some certainty to this complex topic.

The inflammation of the gut is a serious problem in the contemporary world, and the problematic consequences of eating are known. Inflammatory response genes, such as IL1B, IL6, IL8, IL10, and others, are involved in the triggering and maintenance of inflammation through both the genetic and epigenetic mechanisms of action [116]. In particular, NLRP3 inflammasome activation and related IL1-β secretion is a very important endpoint in the inflammation–diet relationship. A potential differential effect of the inflammasome genetic variants in association with the MD has been reported as modulating the risk of Type 2 Diabetes (T2D). In particular, nondiabetic carriers with different NLRP3 genotypes (ref. rs4612666 and rs10733113) differentially changed insulin sensitivity index after 3 years of MD consumption [117].

It is widely known that obesity, already a social/medical problem per se, is related with T2D [118], but only few nutrigenetic studies have described a very close link between specific polymorphism(s), obesity, and T2D risk. Nutrigenetic studies within a cohort of MD consumers showed a close relation between the allelic variants rs7903146 C>T in the Transcription Factor 7-Like 2 (TCF7L2) gene, obesity, and T2D [119]. By analyzing several parameters in multiple complex correlations, including adherence to MD, and by extending the study to other polymorphic T2D-related genes, the authors have obtained a specific “genetic risk score” for each obesity-related allele, and in doing so they have provided a semi-quantitative tool for all owners of risky genotypes to counteract their susceptibility, at least to T2D, by the consumption of specific MD foods. In this regard, it is worth mentioning that, in the MD, the seasonal consumption of legume seeds belonging to the Fabaceae family, rich in bioactive proteins and phytochemicals with properties preventing obesity and T2D, is included [120].

The association between diet quality and telomere length deserves a separate discussion, especially speaking about the nutrigenetics of MD. There is no doubt that, in general, drawing a horizontal line between telomere shortening, ageing, and healthy diets adds value and confirms the ancient and suggestive aphorism that “those who eat well, live longer”. However, it is important to determine that the telomeres are really changed by the effect of a consolidate diet, and it is also fundamental to assess, in each subject, the initial inherited telomere length, as would be done with an allelic variant of any gene. Finally, it is essential to consider which cell or tissue we are talking about to well-correlate the diet effects with the absence or insurgence of specific diseases. A recent study reports telomere length data obtained from the salivary cells of subjects following diets of different overall quality, including the MD. Taking into account the gender and lifestyle differences and also all the possible socio-demographic, anthropometric, and clinical covariates, the authors conclude that a high adherence to MD is associated with a significant lower risk of shorter telomeres. Moreover, it is important to note that analogue results were shown in subjects following other diet regimens, but these data were found significant only for some and not all indexes [121].

Lastly, it is important to consider that, for metabolic serious disorders like obesity, inflammation, and related pathologies, some polymorphisms and genotypes, described in nutrigenetics studies, even important, could be the “iceberg-tip” of the greater integrated systems, often made up of hundreds of genes responsible of the same phenotype. Some alleles are perhaps more described but may not be the most important or representative aspects of the associated phenotype.

### 5.2. The Importance of Considering the Allelic Diversity of CYP2E1 Gene in MD Consumers

A further gene that could be important to correlate with the MD is, in our opinion, CYP2E1. Previously published data of our lab demonstrated that certain CYP2E1 VNTR genotypes (e.g., the A1/A1) might have a protective role against drinking and/or smoking-related cancers [122], and these data show the CYP2E1 gene to be one of the probable advantageous diet-independent genetic actors, promoting the known low cancer rate in the Mediterranean area. Moreover, since some polymorphic alleles of this gene have been described as being implicated in the metabolism of acrylamide [123], a contaminant with genotoxic properties [124] surprisingly discovered in breadsticks and other Mediterranean typical foodstuffs, an allelic discrimination of CYP2E1 is legitimate and desirable, especially regarding the induced hepatotoxicity [125]. Lastly, in more recent times it has been given the statistical evidence that CYP2E1 genotypes, when put in relation with red meat intake, may give a high susceptibility to colorectal cancer in East Asians [126]. It is worth remembering that both red meat and wine were, in the ancient MD tradition, two non-predominant components, but today contrarily their use is increasing, and red meat in particular is consumed not only as part of the principal meals but also in snacks or in bakery products.

### 5.3. The Ability to Assess TAS2R38 Allelic Variants in MD Consumers

It was ascertained that bitter taste perception influences food choices, and in particular it was reported that children with a bitter taste perception, carrying a homozygous TAS2R38 PAV haplotype or a heterozygous PAV/AVI one (ref. to the three polymorphisms: rs714598, rs1726866, rs10246939), have a lower preference for green vegetables like the Crucifera present in Mediterranean area [127]. It is also quite known that the bitter taste in vegetables is associated with the presence of antioxidant molecules [128] with potential protective roles in several diseases, including chronic inflammatory ones. On the other hand, people with a good bitter perception, by not preferring drinking and smoking [129,130] are protected, in percentage terms, from inflammatory and neoplastic diseases induced by these harmful lifestyles. It follows that individuals who are carriers of different TAS2R38 genotypes can be differentially exposed to various genotoxic damages and, at the same time, can be protected by onco-promoter insults.

Moreover, by identifying SNPs associated with taste perception, a recent GWAS pilot study has confirmed the TAS2R38 gene as the most associated with bitter taste at a suggestive level of significance [131]. Thus, the TAS2R38 gene with its alleles, genotypes, haplotypes, and phenotypes is certainly one of the genes that must be considered in a nutrigenetic map, taking also sex and age in account [132], especially in subjects using the MD to whom it can be proposed to enrich their own diet with specific foods with the aim of counteracting such problematic behaviors for a better life expectation. Probably, this latter sentence should also to be applied to other taste genes, since it was recently described that individuals with a greater Body Mass Index (BMI) or waist circumference have a lower taste perception, particularly for salty and sour ones [131]. 

## 6. Most Popular Foods in the World: Substantial Nutrigenomic Differences/Similarities from/to the Mediterranean Diet

Fundamentally, we can assume that the predominant nutrigenomic properties of the Mediterranean diet are due to (i) olive oil polyphenols, (ii) red wine resveratrol, and (iii) tomato lycopene. All these much-studied molecules are mainly responsible for the diet’s known nutrigenomic effects, since they display several anti-cancer characteristics in vitro [133,134,135]. All those regimens that do not admit, or greatly deviate from, the presence of these molecules, could be valuated “at different nutrigenomic benefits” in respect to the MD. However, the complex number of molecules in all the food of a diet regimen can exert several holistic properties on a consumer; thus, it is difficult if not impossible today to valuate clearly the health of a diet, at least in terms of nutrigenomic contribution. We can describe some peculiar foods in the world with the aim to deduce the nutrigenomic distance/similarities from/to the MD.

### 6.1. American Foods

While America’s current populations have dishes and foods similar to Europe, the South American Indios’ diet is particularly stimulating from the nutrigenomic point of view. This population eats, among other things, corn and peanuts and drinks wild grape wine and soft drinks made by boiling ginger, cinnamon, sassafras, and other aromatic herbs in water. A recent on-topic study reported interesting nutrigenomic data regarding the miRNA and histone deacetylase modulation ability of some of these foods and drinks peculiar to these countries with no corresponding examples in the MD. The largely used corn, for example, in the form of corn oil induces a differential profile of the expression of tens of miRNAs related to carcinogenesis in the micro dissected liver of rats [136]. Moreover, the kaempferol, contained in grapes, green tea, and potatoes; the resveratrol of grapes, red wine, blueberries, and peanuts; the sinapinic acid in wine and vinegar; the diallyl disulfide of garlic; and the zerumbone of ginger have recently been considered as “natural HDAC inhibitors” [137] and show an in vitro anti-mitotic effect by regulating miRNAs involved in tumor suppression [138]. Hence, a further motivation to better study these dietary epigenetic regulators, some of them absent in MD, regarding their target specificity to gain potential anticancer dietary epi-adjuvants.

### 6.2. Indian Fruits and Soybean Oil

Some fruits of the Indian popular diet have documented and peculiar nutrigenomic effects. The properties of mango fruit powder on the expression of genes related to fatty acid and glucose oxidation and insulin signaling in the liver and muscle were documented by the increase in the expression of the PPARA and CPT1 genes in an in vivo study [139]. Moreover, it was reported that eating for 1 month a fermented papaya preparation produces a decrease in two biomarkers of occupational stress: the redox balance and heme oxygenase-1 (HO-1) gene expression [140]. No similar effects have been reported today for any MD fruit or food.

## 7. Conclusions and Future Perspectives

The MD, in comparison with the other diet regimens in the world, has all the characteristics of being one of the examples of the “Environment-Livings-Environment” relationship. Similarly, at the biomolecular level, the MD could be described as a patchwork in which several biological and non-biological disciplines are interconnected in a network promoting human health. Moreover, the presence of some genotypes in consumer MD for genes of nutrient metabolism can be considered for a personalized diet that consolidates/corrects healthy/unhealthy habits. 

By remembering that oxidative stress and DNA methylation feature a common denominator, the one carbon cycle [141], we may assume the existence of this chain:

dietary antioxidant → minor ROS → minor DNA damage → normalization of DNA methylation
and the opposite one:
endo/exo-oxidants → greater ROS → greater DNA damage → hypomethylation of DNA for defence

**Figure 2:**
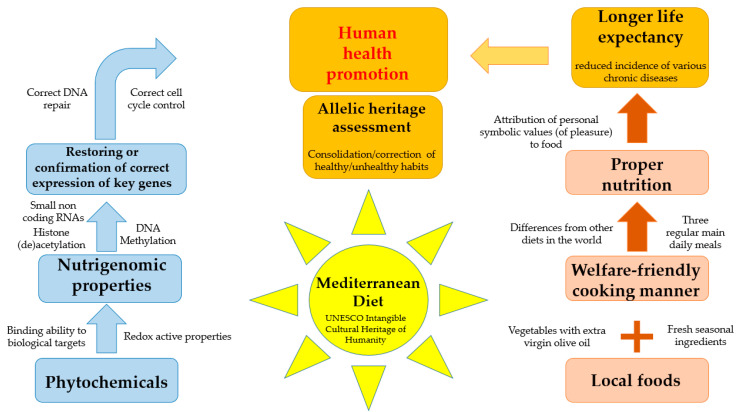
Patchwork expressing the “whole” concept of MD healthy properties. The left block of the diagram shows peculiar biological flowcharts; the right one displays selected non-biological points. The MD, interconnecting with both the blocks and taking advantage of an allelic assessment, represents a network promoting human health.

On the basis of these chains, we can recognize the master strength of the MD: a diet with a high antioxidant and nutrigenomic modulation power. The traditional MD, unlike other diet regimens in the world, provides in the main daily meals of an individual an organized succession of foods and drinks (called first course, second course with side dish, fruit, wine), most of which have a high nutrigenomic value, sometimes summative of more single effects. Thus, a MD consumer daily assumes a wide range of foods, each in smaller quantities, but containing all together a large amount of antioxidants and molecules with nutrigenomic properties (Figure 2).

The recent trend in the world of the agro-foods industry of adding food with beneficial MD molecules (the so-called process of the functionalization of foods) could represent a right way to export the MD and some of its healthy properties also outside the Mediterranean basin. In particular, supplementation with functional food affecting redox regulation may be part of the therapeutic strategy to be considered in the war against the daily work stress of industrial countries [140].

As a future perspective, the consolidation of knowledge in the nutrigenetic/nutrigenomic sense is highly recommended for MD, with the aim of achieving adequate dietary well-being by adopting a nutrigenetic approach. Following any healthy diet, including the MD, without determining the allelic heritage, at least for some known key genes, can help but does not guarantee personalized long-lasting effects and would only be linked to the transient “health effect” of food. Moreover, according to a nutrigenomic approach, the MD can be considered as a world resource. Therefore, knowing the epigenomic effects of some molecules contained in plants, foods, or drinks consumed in the Mediterranean basin could encourage the pharmaceutical manufacturing of specific nutraceutical preparations to be taken as adjuvants even without adhering to MD as a food style.

## Figures and Tables

**Table 1:** Synoptic list of some other typical Mediterranean diet (MD) foods not described in the text with their respective nutrigenomic effects.

MD Food	Peculiar Contained Molecule(s)	Nutrigenomics Effect	Ref.	Notes
Whole meal bread	[Whole food]	Increase of genome-wide DNA methylation.	[90]	In vivo study (Leukocytes) in Caucasian women (Southern Italy) aged 12–87 years).
Rice	[Whole food]	Increase of genome-wide DNA methylation.	[90]	In vivo study (Leukocytes) in Caucasian women (Southern Italy) aged 12–87 years).
Tomato sauce	Lycopene	(a) Free radical-quenching ability. (b) Stimulating antioxidant enzyme activity.	[91]	In vitro study (Wistar rats testis cells).
Parmigiano Reggiano or Grana Padano	β-casomorphin7	Increasing of epigenetic-mediated expression of glutathione S transferase detoxifying enzyme.	[92]	
Pizza	[Whole food]	Increasing of genome-wide DNA methylation.	[90]	In vivo study (Leukocytes) in Caucasian women (Southern Italy) aged 12–87 years).
Peeled raw tomatoes	Lycopene	Anticancer activity related with p53, NF-κB, SIRT1 and with gut microbiome.	[93]	Both male and female animal model studies.
Mushrooms	o-orsellinaldehyde	Indirect inhibition of NF-κb, via IKK-2.	[94]	Present in *Grifola frondosa* Mushrooms.
Asparagus	Asparanin A	Induction of apoptosis via the PI3K/AKT/mTOR pathway.	[95]	Assessed in endometrial cells in vitro and in vivo.
Typical salad: i. Lettuce ii. Onions	Quercetin (3,3′,4′,5,7-pentahydroxyflavone)	(a) Induction of cell cycle arrest, apoptosis, and DNA fragmentation; (b) attenuation of the phosphorylation of MAPK and PI3K/AKT signal proteins; (c) modulation of *CCND1* gene-targeting miRNAs.	[96]	(a), (b) Assessed in VK2/E6E7 and End1/E6E7 human endometriosis cells; (c) Assessed in a mouse model.
iii. Table Olives	Oleuropein; Hydroxytyrosol; Tyrosol.	(a) Anticancer properties; (b) reduction in cyclooxygenase-2 expression.	[97]	Reported in glioblastoma cells.
iv. Capers	Kaempferol	Inhibition of the proliferation of several cancer cell lines via the down-regulation of proteins involved in cancer progression, apoptosis induction, and cell cycle arrest.	[98]	In vitro studies.
v. Fennel	[Whole food]	Significant inhibition of MCF-7 cancer cell proliferation.	[99]	In vitro study with an ethanol extract.
vi. Cherry tomatoes	Carotenoids: lutein, lycopene, and β-carotene	Inhibition of the proliferation of some cancer cell lines at non-toxic concentrations.	[100]	In vitro study on MCF-7, NCI-H460, HeLa, and HepG2 cell lines.
vii. Basil	Methyl cinnamate in essential oil	Cytotoxicity in HeLa, HEp-2, NIH, and 3T3 cell lines.	[101]	
